# Data Challenges With Real-Time Safety Event Detection And Clinical Decision Support

**DOI:** 10.2196/13047

**Published:** 2019-05-22

**Authors:** Eric Steven Kirkendall, Yizhao Ni, Todd Lingren, Matthew Leonard, Eric S Hall, Kristin Melton

**Affiliations:** 1 Department of Biomedical Informatics Cincinnati Children's Hospital Medical Center Cincinnati, OH United States; 2 Department of Pediatrics College of Medicine University of Cincinnati Cincinnati, OH United States; 3 Division of Hospital Medicine Cincinnati Children's Hospital Medical Center Cincinnati, OH United States; 4 James M Anderson Center for Health Systems Excellence Cincinnati Children's Hospital Medical Center Cincinnati, OH United States; 5 Department of Pediatrics Wake Forest School of Medicine Winston-Salem, NC United States; 6 Division of Neonatology and Pulmonary Biology Cincinnati Children's Hospital Medical Center Cincinnati, OH United States

**Keywords:** decision support systems, clinical, clinical decision support, real-time systems, electronic medical records, electronic health records, medical records systems, computerized, informatics, data science, information science, patient safety

## Abstract

**Background:**

The continued digitization and maturation of health care information technology has made access to real-time data easier and feasible for more health care organizations. With this increased availability, the promise of using data to algorithmically detect health care–related events in real-time has become more of a reality. However, as more researchers and clinicians utilize real-time data delivery capabilities, it has become apparent that simply gaining access to the data is not a panacea, and some unique data challenges have emerged to the forefront in the process.

**Objective:**

The aim of this viewpoint was to highlight some of the challenges that are germane to real-time processing of health care system–generated data and the accurate interpretation of the results.

**Methods:**

Distinct challenges related to the use and processing of real-time data for safety event detection were compiled and reported by several informatics and clinical experts at a quaternary pediatric academic institution. The challenges were collated from the experiences of the researchers implementing real-time event detection on more than half a dozen distinct projects. The challenges have been presented in a challenge category-specific challenge-example format.

**Results:**

In total, 8 major types of challenge categories were reported, with 13 specific challenges and 9 specific examples detailed to provide a context for the challenges. The examples reported are anchored to a specific project using medication order, medication administration record, and smart infusion pump data to detect discrepancies and errors between the 3 datasets.

**Conclusions:**

The use of real-time data to drive safety event detection and clinical decision support is extremely powerful, but it presents its own set of challenges that include data quality and technical complexity. These challenges must be recognized and accommodated for if the full promise of accurate, real-time safety event clinical decision support is to be realized.

## Introduction

### Background

All of us as informaticians and, more broadly, users of data realize the common challenges and pitfalls of our substrate. The classic “garbage in, garbage out” and “90% of the time spent working with data is cleaning it” sayings represent some of the most popular conventional wisdom in informatics. Although many of the issues that have popularized these mantras are universal, the context within which the data are created and the context within which the data are manipulated and used often shuffle the focus on particular data challenges and introduce new ones. Challenges in the secondary use of electronic health record (EHR) data or claims data in research are well described [1-5]. This is also the case when considering the use of health data to detect events, particularly when trying to identify safety concerns and errors in a timely fashion.

The continued digitization and maturation of health care information technology has made access to real-time data easier and feasible for more health care organizations. Many EHR vendors have provided some amount of application programming interfaces and other means for real-time access to the data generated by their products, using standards and technologies such as Observational Medical Outcomes Partnership and Fast Health Interoperability Resources [6-9]. The push from many stakeholders for interoperability between EHRs has also helped in this regard. With this increased availability, the promise of using data to algorithmically detect health care–related events in real-time has become more of a reality [10-13]. However, as more researchers and clinicians have utilized real-time data delivery capabilities, it has become apparent that simply gaining access to the data is not a panacea, and some unique data challenges have emerged to the forefront in the process.

### Objective

This brief report serves to highlight some of the challenges that are germane to real-time processing of health care system–generated data and the interpretation of the results. The list generated is by no means exhaustive but does represent some of the most common challenges (and solutions) we have encountered in our experience of designing, implementing, and evaluating numerous real-time event detection systems [14-25]. These challenges were cataloged during the project, highlighted in this report, and augmented with additional challenges from the experiences of the authors in their many other lines of research. Challenges were collected and sorted initially by the first author (EK) and then modified and edited by the other contributing authors. Similar challenges were sorted into high-level challenge categories, and examples from each specific challenge were described.

In this report, we will begin by covering some data issues that are clearly not limited to real-time use and progress to discussing challenges that are more unique to the real-time data use cases. We will offer specific examples of each type and subtype of challenges by referencing a current safety event and error detection project involving the use and joining of data from an EHR and smart infusion pumps and using that data to detect discrepancies between order, administration documentation, and smart pump infusion information. We will (1) briefly summarize the challenge category, (2) describe a specific challenge, and follow this by (3) sharing a specific example of the challenge and, where possible, some mitigation strategies. First, we will give the reader a very brief project overview to set the foundation for the specific examples that follow and to provide a context for the reader to ground in the challenges and examples.

### Project Overview: Fusing Electronic Health Record–Based Medication Ordering, Medication Administration Record, and Smart Infusion Pump Data to Detect Potential Errors

Our research team has demonstrated that EHR data can be used to retrospectively detect discrepancies between how high-risk, continuously infused medications are ordered and how they were documented as being given in the medication administration record (MAR) in the EHR [20-22]. These discrepancies often represent errors, either clinically relevant errors (medication is being given at a rate not intended by the prescriber) or documentation errors (documentation in the MAR is not the dose actually being given). This line of research has evolved and matured to be capable of detecting these discrepancies prospectively, within minutes of the data being available in the EHR. In addition, the retrospective algorithms have been modified to incorporate data from smart infusion pumps, which gives us the capability of discriminating between clinical errors and documentation errors (the pump data are the *source of truth* for what the patient is receiving). The approach relies on chronologically aligning all of the data elements by their respective timestamps. Although simple in concept, in reality this exercise is more difficult than one would anticipate for many reasons. We now describe some of the more salient challenges encountered along this particular line of work; however, they have also been encountered in many other projects relying on real-time and device-related data.

## Results

### Data Challenge Categories, Specific Challenges, and Examples

This report is organized into data challenge categories, specific challenges, and examples. In total, 8 major types of challenge categories are reported, with 13 specific challenges and 9 specific examples detailed to provide a context for the challenges (Table 1).

**Table 1:** Major challenge types, specific challenges, and specific examples related to use of real-time data.

Challenge category	Specific challenge	Example
Selecting the correct data elements	Selecting the right action state and timestamp	Selecting the right action state in medication order data
		Selecting the right timestamp
Other timestamp-related considerations	Delayed documentation—verbal orders	—^a^
	Timestamp conversion and formatting	—
	Visualizing time series data to understand temporal patterns	Raw time data table versus data visualization
Metadata attributes	Misleading (meta)data labels	*Shown to user* data column label
Workflow imprints	Issues that affect performance and capabilities of algorithms	Patient deterioration in the Neonatal Intensive Care Unit necessitates verbal orders
	Delayed action on active orders	—
	Priming pumps: Speeding up infusion pump rates to prime may look like an error, but has no clinical consequence	—
Unstructured data entry	Complexity of human language	Free text dosing of Total Parenteral Nutrition
	Heterogeneity of human language	—
Fusing datasets and the role of device integration	Merging datasets from multiple sources requires valid linking identifiers	The nonintegration of smart pumps with electronic health records
	Clinical decision support blind spots	Use of smart infusion pump drug libraries
Retrospective versus prospective data or detection	Retrospective data and real-time data are processed and accessed differently	The order audit modification issue
Technical versus clinical validity	—	—

^a^Not applicable.

#### Challenge Category: Selecting the Correct Data Element—Multiple Action States and Timestamps Related to Similar Concepts

Modern EHRs contain very elaborate underlying data models, with simple clinical or technical concepts represented in very complex and granular ways. For example, the EHRs that support large health systems with tertiary or quaternary hospitals may have 50 to 100 different data elements that record systolic blood pressure measurements, each with a slightly different context related to workflows. Consequently, related data elements may be scattered across database tables, and despite data dictionaries suggesting relevance, specific data fields may not be populated as expected. Data dictionaries are often incomplete or suboptimal for other reasons, leaving a data consumer to rely on metadata labels and manual inspection of the data to guide accurate selection. Cross reference of the values and patterns between the operational system–derived data (ie, *front-end* views) and the data transformed into reporting databases (usually in a relational database, *back-end* views) is a common approach to guide the stakeholder, but large datasets or closely related data elements can make distinctions challenging and labor-intensive. Multiple locations for similar data may be convenient to both designers and users of the system, but this lack of parsimony can also lead to data completeness issues (reports only pulling data from one source when it should be from both sources) and conflicting values in the EHR (users enter data in multiple locations, and the data conflicts with each other).

##### Challenge: Selecting the Right Action State and Timestamp

Almost all EHR and medical device data are accompanied by one or more timestamps, pieces of data that track the date and time of a particular action or event occurred and were recorded. Although the timestamp denoting that documentation of an event is recorded (*documentation* or *file time*) may be automatically captured, clinicians may also record the time in which events actually occurred or were documented to have occurred (*action time*). Subsequent documentation may amend the originally documented action time, but each amending action would also generate a corresponding timestamp representing when the amendment was recorded (*amending time*). In addition, many data representations of clinical concepts or actions have multiple action states. Selecting the correct action state (including or excluding action states) and the right timestamp field can be difficult. For action states, it is not uncommon to have many closely related or overlapping enumerated list items that can make this selection complicated. For each of these action states that are recorded, there may be several timestamp fields to choose from. Selecting the wrong action state or timestamp can lead to misleading inferences from the data. Mitigation strategies for this challenge type include careful evaluation of all possibly relevant data fields related to the data request, reviews of existing data dictionaries, and careful analysis and validation of the output from data discovery activities. This analysis and validation should be from both the technical analyst and the clinical subject matter expert.

##### Example: Selecting the Right Action State in Medication Order Data

Poor communication between data requestors and data provisioners may lead to confusion between selecting and including order data (how things were ordered) versus MAR documentation data (how orders were acted or not acted upon) for use in reports or algorithms. In real clinical practice, not all medication orders are carried out for a variety of reasons and one should not assume that a medication order means that a patient actually received the medication. An action state of *Given* or *Not Given* in the MAR is more indicative of the clinical actions that actually took place. Smart infusion pumps generally log each action that a pump undergoes, whether it is an automated procedure or the result of user programming. Smart pumps may support dozens or hundreds of action states, with the most frequent states logged including *infusion started*, *infusion stopped*, and *infusion restarted*. If an analyst or researcher is attempting to count the number of times an infusion moved from an inactive (not infusing) to an actively infusing state and only counts the *infusion started* state, they will underestimate the count by missing the *infusion restarted* state (assuming the restarted state is not accompanied by logging an *infusion started* action simultaneously). This can obviously affect denominators in rate metrics. In one project centered around risk-stratifying and detecting acute kidney injury (AKI), early iterations of an algorithm based on medication profiles overestimated the population at risk for AKI because we incorporated medication orders and not medication administration data. Not all orders are acted upon and administered to patients. The accuracy of the algorithms improved with modification.

##### Example: Selecting the Right Timestamp

MAR documentation typically records a medication action state and several timestamps pertaining to the action state. The two most common are an action time and a documentation or file time. In many workflow instances, the time between the two may be trivial. In busy workflows or high clinical throughput scenarios, documentation of the action state may occur much later (eg, *back-documentation* by a nurse) and the interval between the 2 timestamps could be substantially large. The main challenges when utilizing timestamps in real-time applications are twofold: (1) interchanging the 2 values can affect time interval measurements by causing under- or overestimation inaccuracies and (2) back-documentation has the potential to undermine the power of real-time detection by delaying the availability of data. In the Medication Safety project described above, the delaying of documentation of MAR actions delays the ability of our algorithms to detect discrepancies between orders and actual administration, thereby limiting the ability to identify errors in real-time. In effect, a blind spot is created by the lack of timely documentation. Allowing for an appropriate time window to accommodate back-documentation is challenging because too wide a window enlarges the blind spot of vulnerability for prolonged time periods. However, establishing too narrow a window may increase false positive real-time error rates caused by delayed documentation. Understanding workflows is critical in fine-tuning the optimal tolerance for delayed documentation, which may vary from one unit or institution to another.

#### Challenge Category: Other Timestamp-Related Considerations

Timestamp data are central to the concepts discussed in this report, especially when merging and synchronizing disparate datasets. Even data representing a universal concept, such as time, can take on many forms that make processing it appropriately and making accurate inferences a challenge and is an exercise that is prone to error and limitations. The following are the specific challenges and examples related to timestamp data.

##### Challenge: Delayed Documentation—Verbal Orders

Verbal orders, by definition, are orders that are not documented in the EHR or other associated systems. Safety goals and regulatory mandates state that verbal orders be kept at a minimum, and in most cases, the expectation is that verbal orders be documented electronically at some point after they are clinically acted upon. This lag in time between the time they are carried out clinically and the time that they are documented electronically (if at all) undermines the capabilities of algorithms that depend on timely data streams to be functional. Technical issues that lead to delayed delivery of data can have the same effect. In many cases, this may lead to a false positive event detection, lowering the performance characteristics of an algorithm and leading to decreased confidence and buy-in from the recipients of the algorithm output. Mitigation strategies include allowing for a reasonable time window for verbal orders to appear. In our project, we allow 30 min for the new documentation of a verbal order to appear in the EHR and data feeds. Data from this allowance would then negate, or *call off*, the event detection and notification.

##### Challenge: Timestamp Conversion and Formatting

**Figure 1:**
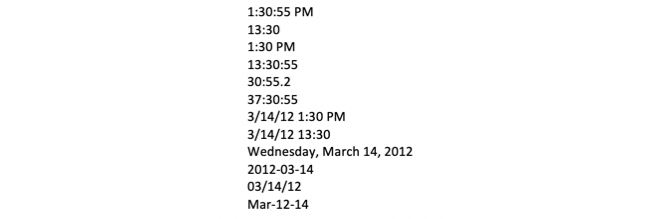
Time and date represented in many different formats.

**Figure 2:**
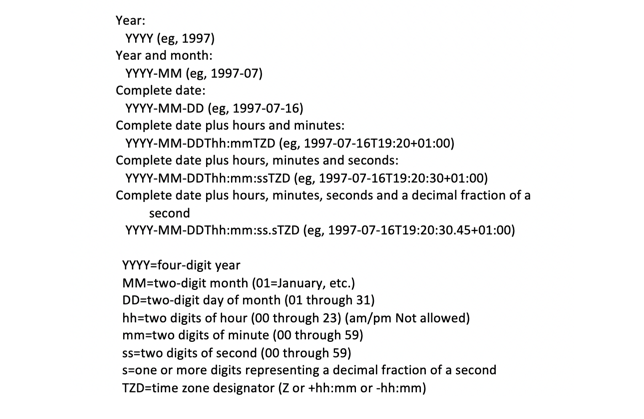
Representation of different amounts of granularity and specificity in timestamps, ranging from year only to fractions of a second, and including timezone information. Content from the World Wide Web Consortium.

Timestamp data can be represented in many different formats. A thorough discussion of timestamps and their many challenges is out of scope for this report, but Figure 1 demonstrates a couple of different formats in which dates and times can be represented. Granularity and specificity of the timestamp is also an issue, as shown in Figure 2 [26]. Converting to an unexpected format during the extract-transfer-load (ETL) process (eg, converting YYYY-MM-dd HH:mm:ss to YYYY-MM-dd only) would cause unexpected errors in system outputs and subsequently pose a challenge to algorithm debugging. Other complicating factors include accounting for Daylight Saving Time adjustments, the selection of an epoch to count the duration of time (Unix-based systems use the time elapsed since 00:00:00 Coordinated Universal Time [UTC], Thursday, January 1, 1970), and the conversion of one time format to another (eg, from Eastern Standard Time to UTC). In particular, simple mistakes with timestamp entering and formatting, especially when fusing data from manually input sources, can cause pairing errors and have important negative effects in system processing. ISO 8601, drafted by the International Organization for Standardization (ISO), is intended to be the international standard for representation of dates and times but not all systems adhere to this representation [27].

##### Challenge: Visualizing Time Series Data to Understand Temporal Patterns

Working with raw data or simple nonvisual outputs of the data (such as in the tabular format) may be the simplest way to review time-based data, but it does not lend itself to appreciating chronologic events and judgements about the interval between 2 data points easily (eg, intervals such as elapsed time). This is usually most easily accomplished by a visualization, such as a timeline or other graphic representations, where one axis is linear time. Without some representation of the interval magnitude, reviewers will need to exert more cognitive effort to appreciate these measures. To mitigate this issue, we recommend the use of robust visualization analytics tools to appreciate these patterns. The following simple example demonstrates the utility of even rudimentary tools.

##### Example: Raw Time Data Table Versus Data Visualization

A comparison below demonstrates the 2 different data representation methods for the same dataset. In this example, data from several data element categories are represented in a tabular format and time series format.

**Figure 3:**
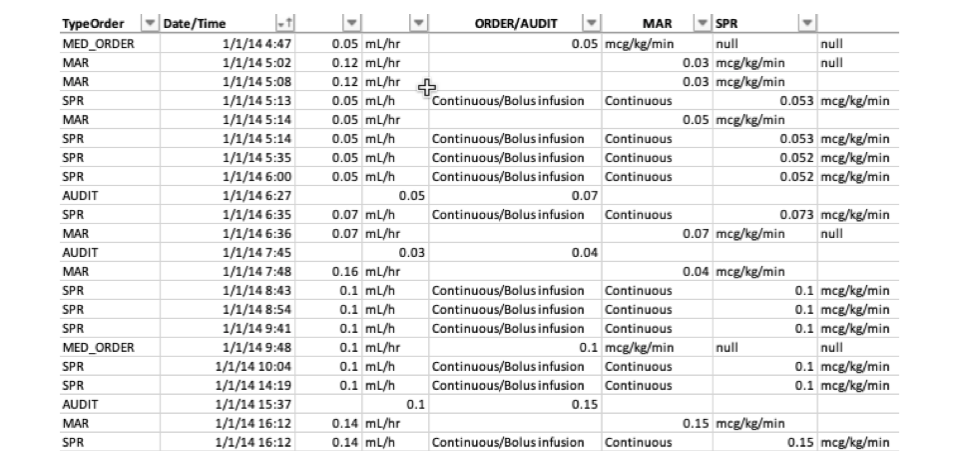
Tabular representation of the medication order, medication administration record (MAR), and smart pump record (SPR) data.

**Figure 4:**
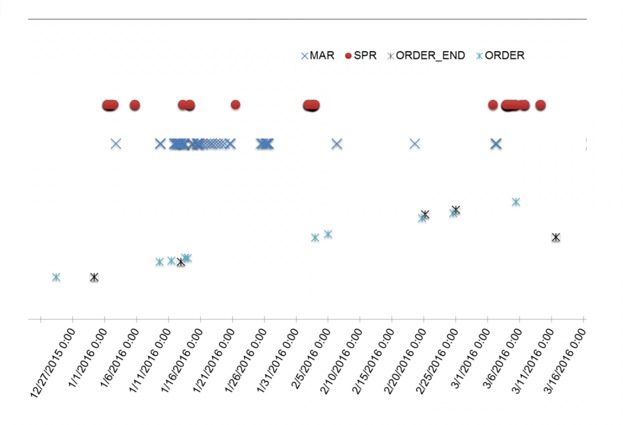
Graphic visualization representation of the medication order, medication administration record (MAR), and smart pump record (SPR) data in time-series format.

As shown in Figures 3 and 4, it is much more efficient to interpret time-series data in a visual format, which allows one to quickly understand the magnitude of events represented by the data, such as the time between events or overall relationship of several events. It also allows easier representation of a high number of events or occurrences by creating overlays for each event type of interest.

#### Challenge Category: Metadata Attributes

##### Challenge: Misleading (Meta)data Labels

EHR systems that generate data often do so for a primary purpose that is not research related and/or not with a priority on using precise, accurate, and nonoverloaded data labels. Written another way, clinical information systems are not usually designed and implemented with secondary uses of data as a priority. Data consumers are often left to guess about how the data are generated, based on the metadata information in data dictionaries (if available), database structures, attribute titles/description, and patterns within the data values. Imprecise or inaccurate metadata labels can lead to incorrect assumptions and interpretations. Resolution of this lack of (or missing) metadata requires both the technical analyst and clinical subject matter expert to work collaboratively to understand the data. In some cases, our teams have had to run simulations in sandbox environments of proprietary software and examine output to understand how the data are being generated by the system.

##### Example: Shown to User Data Column Label

Ambiguous metadata labels can be misleading. A binary data field (such as Boolean values) with a field name of *Shown_to_User* in a clinical decision support (CDS) database may lead a data consumer to guess that the field represents whether or not users of the system were exposed to CDS, for example, whether or not a user saw an alert. Data inspected after some reverse-engineering exercises involving simulation in a nonproduction environment, however, reveals that the data field represents a configuration setting (ie, whether the alert should be shown to user). This was discovered after running simulations in which the users conjure the alert in a scenario, but the Shown_to_User data for the corresponding records did not convert from a FALSE value to TRUE. Closer inspection reveals that the default setting for the alert is to not be shown to the user (Shown_to_User is FALSE) and that the data field is static (after viewing CDS, the data remain set to FALSE instead of converting to a TRUE value). Previous analyses using the Shown_to_User data have potentially been inaccurate, and any inferences based on this false assumption can be latently wrong and hard to detect.

#### Challenge Category: Workflow Imprints—Issues That Affect Performance and Capabilities of Algorithms

Appreciation of workflows is paramount to any clinical studies, and the same can be said for clinical informatics research with data generated by operations-supporting software and hardware. Unique clinical workflow idiosyncrasies can strongly affect the performance of real-time CDS algorithms and applications. These workflows can manifest in obvious and subtle ways in the data. The former is easy to detect but may or may not be easy to adjust for. The subtler cases, much like the metadata example above, can be harder to notice and accommodate for. As workflow is a universal and heterogenous concern, we will present several challenges and examples below. To minimize this challenge, data stakeholders must understand both the clinical workflows that generate the data and the technical systems that manipulate and store the data.

##### Example: Patient Deterioration in the Neonatal Intensive Care Unit Necessitates Verbal Orders

In the intensive care unit (ICU) environment, the clinical condition of patients can change quickly and abruptly. As such, medications are frequently ordered and administered in patterns that do not match the usual pattern of *order-then-administration*. For instance, in the case of a rapidly declining patient who needs more medication for blood pressure support (eg, an ionotrope), a physician or other prescriber may ask the bedside nurse to increase the dose of an already infusing medication before placing an electronic order for the rate in the EHR. This may happen because the prescriber is tending to other bedside needs in a critically ill patient and does not want to step away to place or document the order. The nurse may therefore document the MAR before the order which, in our algorithms, may be considered an erroneous dose as no electronic record of the order exists yet. To accommodate this challenge, we allow for a 30-min lag after the MAR is documented before calling the administration an error. This 30-min window is *waiting* for a verbal order to be documented (within a reasonable time frame) before triggering a notification that a potential error has occurred. This creates a trade-off between the *real-timeness* of the algorithms and the desire to decrease false positive error calls and notifications.

##### Challenge: Delayed Action on Active Orders

Total parenteral nutrition (TPN) is an intravenous nutrition alternative given to patients who cannot tolerate normal feeding, orally or through feeding tubes. It is a complex compounded liquid that takes time to prepare. TPN orders are often placed early in the day, with the knowledge that the new solution will not be administered until afternoon or evening. The rates on the orders, however, may be active at the time the order is placed and be changed over the day. Algorithms that use TPN data must take this factor into account and allow for a delayed action on the otherwise (technically) active order.

##### Challenge: Priming Pumps—Speeding Up Infusion Pump Rates to Prime May Look Like an Error But Has No Clinical Consequence

Before administering intravenous medications via an infusion pump, bedside staff must often *prime* the pump equipment by filling the lines/tubing with the medication substance to remove air and provide consistent delivery of the drug. The need to do so efficiently often leads to adjusting pump rates to high levels to accomplish priming quickly. This may manifest in the data as apparent erroneously high rates of infusion if there is no tagging of the priming action or other record in the data. Bolusing a medication (an intentional brief high rate of delivery of medication to the patient) over an already continuously infusing rate can also mislead data consumers to falsely believe an error has occurred. Inquiries to clinicians about their workflows in relation to the data patterns observed will often explain the digital manifestations and require refinements to real-time systems to account for these anomalies.

#### Challenge Category: Unstructured Data Entry

Approximately 30% to 50% of data entries useful for quality improvement are available only in an unstructured text format in modern EHRs [28]. The importance of this information has gained increasing recognition for quality improvement and patient safety. In the medication safety project described above, our clinicians often use both structured computerized provider order entries and free-text communication orders to prescribe allowable dose adjustments and ranges. As a best practice across the institution, the clinicians are encouraged to make dose adjustment via structured entries. However, the continuous infusion medications usually include instructions for frequent and complex dose changes related to the physiologic state of the patient. In this regard, the existence of free-text orders are artifacts that reflect the dynamic and changing clinical status of typical critical care patients. To identify information embedded in unstructured narratives, natural language processing (NLP) has become a critical component of computerized clinical support. Nevertheless, the complexity and heterogeneity of the human language makes its application and dissemination a challenging task. Conversion of unstructured data to structured data, when possible and favorable, minimizes this challenge.

##### Challenge: Complexity of Human Language

Different from structured entries that are enumerable, the complexity of human language creates an infinite space with countless linguistic variants. Accounting for the variants with NLP is therefore an onerous task.

##### Example: Free Text Dosing of Total Parenteral Nutrition

For instance, a physician could specify a dose adjustment explicitly as, “Please decrease TPN to 10 mL/hr”. They could also specify the dose adjustment implicitly as, “Please decrease TPN rate so that TPN rate + feeding rate=12 mL/hr”. In addition, modifiers are commonly used to adjust one’s meaning. For example, the physician could specify, “When the new bag arrives, please decrease the rate to 10 mL/hr”, suggesting that the rate is prescribed for when the next medication supply bag is delivered to the bedside. To accommodate language complexity, researchers tend to use flexible language constraints (eg, loose regular expressions) to parse narrative content. To address this challenge, we have evolutionarily modified our algorithms as we have identified false negatives and false positives and have found that necessary changes have decreased in frequency over time. Nevertheless, edge cases are identified in an ad hoc manner and require manual inspection constantly.

##### Challenge: Heterogeneity of Human Language

Although it is rarely noticed in single-institution applications, heterogeneity of the human language has been recognized as a major barrier to the dissemination of NLP-integrated CDS tools across health care institutions. For instance, the free-text narratives from a pediatric health care institution would primarily describe signs, symptoms, procedures, and medications for pediatric patients, whereas the narratives from a general hospital would primarily describe those for adult patients. The language heterogeneity therefore greatly affects the performance of CDS tools delivered from one institution to another. Customization is necessary for using NLP tools developed from external institutions and it requires both domain expertise and intensive resources.

#### Challenge Category: Fusing Datasets and the Role of Device Integration

##### Challenge: Merging Datasets From Multiple Sources Requires Valid Linking Identifiers

Many medical devices currently in use in health care facilities are not fully integrated in a *closed-loop* manner with EHRs and other systems, meaning that they are often not fully interoperable.

##### Example: The Nonintegration of Smart Pumps With Electronic Health Records

Infusion pump programming errors may result in a combination of missing patient and/or drug identifiers. For example, a patient’s medical record number (MRN) or encounter ID may be mis-entered or bypassed altogether. Similarly, a basic infusion rate may be selected without specifying the medication being administered. In the event of missing or incorrect patient or medication identifiers, linking smart pump generated data with order logs or MARs, particularly in real-time, becomes vastly more complicated. This is particularly true when the administered drug is commonly used and may have been ordered for several patients concurrently, which makes inference by the time of administration difficult. In our previous evaluation of NICU smart infusion pumps, we found in a convenience sample that although 89% of pump records included the medication ID, 76% contained a valid patient ID and only 68% contained both valid patient and medication IDs. Fortunately, only 3% were missing both identifiers (Table 2). Both data elements must be valid to accurately and efficiently link EHRs and infusion pump data.

##### Challenge: Clinical Decision Support Blind Spots

Almost all forms of CDS depends on some data elements to be present to drive the rules engines underlying the CDS platform. Lack of that data leads to unavailability of CDS and lost opportunities to inform and optimize treatment decision making.

##### Example: Use of Smart Infusion Pump Drug Libraries

Reviewing of the smart infusion pump-related literature reveals that one of the largest purported benefits of using smart pumps is to perform medication dose and administration checking at the time of pump programming by a bedside user. Smart pumps use drug libraries with various rules around allowable doses and rates of administration. However, use of the libraries requires, at a minimum, knowledge about what drug is about to be infused so it *knows* what drug library rules to enforce. A frequent workaround to programming this information into the pump is to use a generic drug profile, which (1) does not label the data with any kind of clear medication identifier and (2) effectively renders the potential CDS unusable, as the rules cannot be invoked. This leads to a CDS blind spot.

#### Challenge Category: Retrospective Versus Prospective Data/Detection

##### Challenge: Retrospective Data and Real-Time Data are Processed and Accessed Differently

There are fundamental differences in both how one accesses and processes data depending on, among other factors, whether it is coming from a data repository such as a relational database or from live data feeds from the operational source that is generating the data (such as via a real-time data interface). Retrospective data sources have frequently been subject to many ETL operations, any of which can and usually do alter the data in some fashion. These alterations are not always clear, and byproducts can arise in the data which are hard to detect and account for. ETL operations are also opportunities for errors to arise, many of which can be silent and lead to issues such as incomplete data extractions and incomplete datasets.

**Table 2:** Distribution of smart infusion pump data records with valid medication and patient IDs.

Medication ID	Patient ID, n (%)	
	Present	Missing
Present	5440 (68)	1680 (21)
Missing	640 (8)	240 (3)

##### Example: The Order Audit Modification Issue

A pattern of medication data was uncovered during retrospective data review that demonstrated the perils of taking data at face value. When an order was modified (recorded as an audit), the new value overwrote the original value. However, the data user viewing the data as an individual record would not notice this pattern and assume the value presented was the original value. The data quality issue would also not be noticed when analyzing the data in aggregate (such as performing descriptive statistics on doses ordered using the data). Real-time data interfaces would pull the data in real-time and send it for processing, which would mitigate the concern of using erroneous data. Review of these data obtained via retrospective reports (pulled from a data repository) would have different values from data obtained in a real-time, prospective manner but the difference would be extremely difficult to identify under normal inspection procedures. Only with rigorous examination would the issue be uncovered. We have applied postprocessing *patches* to our datasets to accommodate for ETL-related idiosyncrasies that we have noted. These patches revert the data to a state more reflective of reality.

#### Challenge Category: Technical Versus Clinical Validity

A primary purpose of real-time error detection should be to mitigate patient’s exposure to harm. Detected errors which do not support that purpose should not necessarily be reported in real-time owing to their limited utility. For example, an error may be predictably detected based on improper documentation in which the correct dose was administered but the clinicians did not follow standards of practice in timely documentation of medication use (eg, placing an electronic order after a prolonged period of time following the corresponding verbal order). Although the detection algorithm performed as expected based on documentation, these errors have no potential to cause harm and are not clinically consequential. Similarly, overly sensitive algorithms may detect dosing administration errors that are technically incompatible with medication orders—though the dosing discrepancy is clinically meaningless and presents minimal risk of causing harm. An example would be a dosing discrepancy error where the dose given exceeds the prescribed dose by 1% or 2%. For almost all medications, this *overdose* is not clinically significant. To avoid inundating clinicians with unactionable alerts, some tolerance should be included in error detection algorithms to accommodate true errors that have little or no potential for clinical impact. A final example of an uninformative though technically accurate alert occurs when repeat errors are detected for the same underlying, persistent but inconsequential event.

## Discussion

### Practical Implications

In this paper, we have compiled many challenges unique to using real-time data. Most of the enumerated challenges and examples in this paper will be familiar to many data stakeholders, from requestors to the technical analysts who work diligently to provision complete, accurate datasets. The challenges were presented in both high-level overviews and grounded in specific examples, but the principles behind them are generalizable to almost any kind of data-based work. The different challenges should be seen as equally important as a standalone list, but some will be more important to monitor and address than others, depending on the use case and types of data involved. Various mitigation strategies were presented here, but the commonality among most of them is that to be effective, they must generally be built on a foundation of multi-disciplinary teamwork. These teams need to be collaborative in nature and be composed of members with strong data science knowledge, biomedical informatics skills, and familiarity with the clinical processes and workflows that are at the heart of the project in progress. As with any challenging process, these efforts take time and resources; this should be factored into any timelines and planning as appropriate. We typically recommend adding a time buffer to accommodate this in the initial stages of a project, as well as allocation of some resources for maintenance once the project is in a sustain phase. The systems that create and pass along the data are in constant flux—so too should be the vigilance of the downstream systems utilizing the data.

It should be noted that our list here should be seen as a starting point—certainly others in the field could easily add many other types of categories and examples from many different domains of the digital health domain and beyond. This report was meant to start the conversation and bring recognition to the fact that working with real-time data is not as easy to do practically as it is conceptually. This represents the first crucial step.

As with an early work, there are many ways we can envision advancing our knowledge in this area. Furthermore, research in these unique data challenges could focus on formalizing the list further and specifying the importance of each in different contexts. It would be particularly helpful if future investigators could continue to report on best practices and strategies of how to address specific challenges, as well as develop a systematic framework to assist in avoiding the issues in the first place or mitigating them once they have occurred.

Data are a powerful tool—but we must recognize and promote best practices if we are to get the most value out of data and not derive false insight from our work.

### Conclusions

The use of real-time data to drive safety event detection and clinical decision support is extremely powerful but presents its own set of challenges including data quality and technical complexity. These challenges must be recognized and accommodated for if the full promise of accurate, real-time safety event CDS is to be realized.

## Abbreviations

AKI: acute kidney injury
CDS: clinical decision support
CPOE: Computerized Provider Order Entry
EHR: electronic health record
ETL: extract-transfer-load
ICU: intensive care unit
ISO: International Organization for Standardization
MRN: Medical Record Number
MAR: Medication Administration Record
NLP: natural language processing
NICU: neonatal intensive care unit
TPN: total parenteral nutrition
UTC: Coordinated Universal Time
